# Potential Metabolite Biomarkers for Early Detection of Stage-I Pancreatic Ductal Adenocarcinoma

**DOI:** 10.3389/fonc.2021.744667

**Published:** 2022-01-19

**Authors:** Yingying Cao, Rui Zhao, Kai Guo, Shuai Ren, Yaping Zhang, Zipeng Lu, Lei Tian, Tao Li, Xiao Chen, Zhongqiu Wang

**Affiliations:** ^1^ Department of Radiology, Affiliated Hospital of Nanjing University of Chinese Medicine, Nanjing, China; ^2^ Pancreas Center, The First Affiliated Hospital of Nanjing Medical University, Nanjing, China; ^3^ Department of Pathology, The First Affiliated Hospital of Nanjing Medical University, Nanjing, China

**Keywords:** biomarker, serum, early diagnosis, pancreatic cancer, metabolomics

## Abstract

**Background & Objectives:**

Pancreatic ductal adenocarcinoma remains an extremely malignant tumor having a poor prognosis. The 5-year survival rate of PDAC is related to its stage (about 80% for stage I vs 20% for other stages). However, detection of PDAC in an early stage is difficult due to the lack of effective screening methods. In this study, we aimed to construct a novel metabolic model for stage-I PDAC detection, using both serum and tissue samples.

**Methods:**

We employed an untargeted technique, UHPLC-Q-TOF-MS, to identify the potential metabolite, and then used a targeted technique, GC-TOF-MS, to quantitatively validate. Multivariate and univariate statistics were performed to analyze the metabolomic profiles between stage-I PDAC and healthy controls, including 90 serum and 53 tissue samples. 28 patients with stage-I PDAC and 62 healthy controls were included in this study.

**Results:**

A total of 10 potential metabolites presented the same expression levels both in serum and in tissue. Among them, a 2-metabolites-model (isoleucine and adrenic acid) for stage-I PDAC was constructed. The area under the curve (AUC) value was 0.93 in the discovery set and 0.90 in the independent validation set. Especially, the serum metabolite model had a better diagnostic performance than CA19-9 (AUC = 0.79). Pathway analysis revealed 11 altered pathways in both serum and tissue of stage-I PDAC.

**Conclusions:**

This study developed a novel serum metabolites model that could early separate stage-I PDAC from healthy controls.

## Introduction

Pancreatic ductal adenocarcinoma (PDAC) is one of the most fatal malignancies. It’s estimated that there were 420,000 new pancreatic cancer cases and 410,000 pancreatic cancer deaths worldwide in 2020 (1). Currently, surgical resection is the most important clinical treatment for PDAC patients, whereas about 80%~85% of patients are in advanced stages at the time of initial diagnosis (2, 3). The 5-year overall survival rate of PDAC is only 9%, but the survival rate of I stage PDAC can be as high as 80% (2, 4, 5). Thus, detection at the early stage is the key to improve the prognosis of PDAC.

To date, conventional methods for pancreatic cancer detection mainly include multidetector computed tomography (CT) and the serum level of carbohydrate antigen 19-9 (CA19-9) (6). However, CT is limited in the detection of small and isoattenuating PDAC (7). CA19-9 is likely to produce many false positives results among other nonmalignant diseases, such as chronic pancreatitis and liver cirrhosis (8, 9). Hence, there is an urgent clinical need to develop a reliable method for the detection of PDAC at an early stage.

Metabolomics is a novel technology, which is defined as a holistic analytical approach to identify all the low molecular weight metabolites in an organism or cell system (10, 11). The serum is a non-invasive and easily available source of metabolic samples in the clinic. Therefore, serum metabolic analysis between PDAC and healthy controls has been widely reported (12). However, most previous studies ignored the bias brought by the stage of PDAC, which may limit the utility of metabolite biomarkers in diagnosing PDAC patients at an early stage. In addition, most serum metabolites are lack specificity for PDAC (13). Tissue metabolism can provide more systematic metabolic information, which is helpful to explore the upstream regulation mechanism of PDAC and search for more specific biomarkers (14). Currently, tissue metabolic analysis for early PDAC is rather little.

Therefore, this study aimed to identify reliable metabolic biomarkers for stage-I PDAC based on tissue and serum samples using an untargeted metabolomics method. Finally, a clear distinction between stage-I PDAC from healthy controls (HCs) was detected, and a metabolites-based model that have potential value for early screening of PDAC was constructed.

## Materials and Methods

### Patients and Study Design

In this study, the paired PDAC serum and tumor tissue sample in the discovery set were enrolled from the Affiliated Hospital of Nanjing University of Chinese Medicine and the First Affiliated Hospital of Nanjing Medical University. Discovery set included 66 serum samples and 53 tissue samples: 17 serum samples from stage-I PDAC, 49 serum samples from HCs, 13 paired stage-I PDAC and distal noncancerous tissues (DNTs) samples, 27 unpaired DNTs samples from other stage-II PDAC. The serum samples in the external validation set were from the Jinling Hospital. The validation set included 24 serum samples: 11 from stage-I PDAC and 13 from HCs. Serum and tissue samples of participants were recruited between January 2019 and December 2019. This prospective study obtained the approval from the independent Ethics Committee of Affiliated Hospital of Nanjing University of Chinese Medicine, the First Affiliated Hospital of Nanjing Medical University, and Jinling Hospital, respectively. All patients had signed informed consents. All PDAC patients in this study were new cases at the I stage and confirmed by pathological examinations.

Exclusion criteria of PDAC patients were as follows: 1) the enrolled patients had received any radiology or chemotherapy treatment before enrollments; 2) patients with hematological diseases or immune blood system disease; 3) patients with another tumor history. 4) patients with cancer recurrence. Exclusion criteria of HCs were as follows: 1) participants with hematological diseases or immune blood system disease recently; 2) participants with any tumor history. The medical history, such as diabetes and cardiovascular diseases, and data of tumor biomarkers were also collected.

### Data Collection and Histopathological Examinations

Demographic information, clinical features and laboratory testing were collected at the time of diagnosis. Demographic information included gender and age; clinical features included diabetes, hypertension, and smoking history. Laboratory testing included the serum level of carbohydrate antigen 19-9 (CA19-9).

All the PDAC tissue specimens were frozen intraoperatively and further sliced and scored for histology. All PDAC samples in this study were new cases at the I stage and confirmed by pathological examinations. Each tissue samples were divided two part: a part was fixed in 0.1%glutaraldehyde and 2%paraformaldehyde in phosphate buffer for electron microscopy and immunocytochemistry analysis, and another part was immediately stored in liquid nitrogen for a short time, and then stored at -80°C till metabolomics analysis. Histopathological information included tumor location and size, tumor grading and TNM tumor stage according to the 8th edition of the American Joint Committee on Cancer (AJCC) staging system. Histopathological information included tumor location and size, tumor grading and TNM tumor stage according to the 8th edition of the American Joint Committee on Cancer (AJCC) staging system.

### Sample Preparation

Each patient provided 5ml of fasting blood between 8 am and 9 am, before the operation and any treatment; each healthy control provided 5ml of fasting blood samples between 8 am and 9 am. Serum samples were stored for 1h at room temperature and then centrifuged for 10 min at 4°C and 4,000 rpm. The samples were separated into 200 µL microtubes and stored at a -80°C. The serum metabolites were extracted by adding 400 µL of methanol/acetonitrile to 100 µL of serum samples. Then, the sample was vortexed and cooled at -20°C for 30 min. The sample was then centrifuged at 14,000 rpm for 20 minutes at 4°C. The supernatant of each sample was collected and stored at -80°C for analysis.

A piece of the tissue (10 mg) for each sample was mixed with 1.0 mL methanol/acetonitrile into microtubes. The tube was vortexed, ultrasonicated for 30 minutes, and cooled for 1 hour. The tube was then centrifuged at 14,000rpm for 20 minutes at 4°C. The supernatant of each sample was collected and stored at -80°C for analysis.

Quality Control (QC) samples were pooled by mixing all extracted serum or tissue samples to ensure the repeatability and stability of the metabolomics. The pretreatment of QC samples was consistent with the study samples.

### Metabolomics Analysis

UHPLC-Q-TOF-MS was used as a metabolites separation and detection platform to identify the untargeted metabolite profiling of serum and tissue in the discovery set. An Agilent 1290 Infinity UHPLC HILIC (100mm*2.1mm, 1.7um, Waters) was used for chromatographic separation. The mobile phase was composed of solvent A, 0.1% (volume fraction) formic acid in water, and solvent B, 0.1% (volume fraction) formic acid in acetonitrile. The gradient program of serum samples was as follows: 0-1min 95%B, 1-14min 65%B, 14-16min 40%B, 16-18min 40%B, 18-18.1min 95%B, 18.1-23min 95%B. The gradient program of tissue samples was as follows: 0-0.5min 95%B, 0.5-7min 65%B, 7-8min 40%B, 8-9min 40%B, 9-9.1min 95%B, 9.1-12min 95%B. The flow rate was 0.3 mL/min and the column oven was set to 25°C. The serum data was collected under the positive and negative ion modes of Triple TOF 5600 ^+^mass spectrometer. Electrospray ionization (ESI) experiment source conditions were as follows: Ion source gas 1, 60 psi; ion source gas 2, 60 psi; curtain gas,30 psi; source temperature, 600°C; ionspray voltage floating, +5000V and -5000V. The tissue data were collected under the positive and negative ion modes of Agilent 6550 ^+^mass spectrometer. Electrospray ionization mass spectrometry (ESI-MS) experiment source conditions were as follows: nebulizer pressure, 20 psig; drying gas, 16L/min; gas temperature, 400°C; Capillary voltage, 3000V; nozzle voltage, 0V; fragment voltage, 175V; The mass scan rate was 50-1200m/z. Information-dependent acquisition (IDA) was performed to detect and identify MS spectra. The collision energy was +50V and -20V. The declustering potential was +60V and -60V. Moreover, QC samples were inserted into the analytical sequence randomly and analyzed five times.

GC-TOF-MS was used as a targeted platform to quantify the identified biomarkers in the validation set. Agilent DB-WAX capillary column (30mm*0.25mm ID*0.25um) was used for chromatographic separation. The initial temperature was maintained at 50°C, then increased to 220°C for 5 minutes. The carrier gas of this system was helium (1.0 mL/min). QC samples were set to monitor the repeatability and stability of the system. Agilent 7890 gas mass spectrometry conditions were as follows: Injection temperature was 280°C; ion source temperature was 230°C; transmission line temperature was 250°C. The energy in electron impact mode was 70eV. MSD ChemStation software was used to extract the chromatographic peak area and retention index. All the samples were kept at 4°C during analysis.

### Data Statistics and Analysis

The raw data were imported to XCMS to perform peak extraction, peak matching, retention time correction. Variable distribution was normalized using Log2 transformation and Pareto scaling for all data. Then the data was imported into SIMCA-p software for multivariate statistical analysis, including principal component analysis (PCA), partial least squares discrimination analysis (PLS-DA), and orthogonal least squares discrimination analysis (OPLS-DA). Two hundred permutations tests were used to test the reliability of the models. Metabolites were further applied to the univariable analysis, including Student’s t-test and Fold change analysis. The criterion of differential putative metabolites was variable importance in the projection (VIP) > 1 and *P* < 0.05. The logistic regression model was built to identify the differential metabolites based on clinical factors. The receiver operating characteristic (ROC) curve was performed to evaluate the diagnostic ability of differential metabolites and the metabolic model using GraphPad Prism statistical software. Hierarchical clustering analysis and pathway enrichment analysis were performed by the MetaboAnalyst website. Pathway analysis based on “Kyoto Encyclopedia of Genes and Genomes” (KEGG).

## Results

### Demographic Characteristics of the Study Population

**Figure 1:**
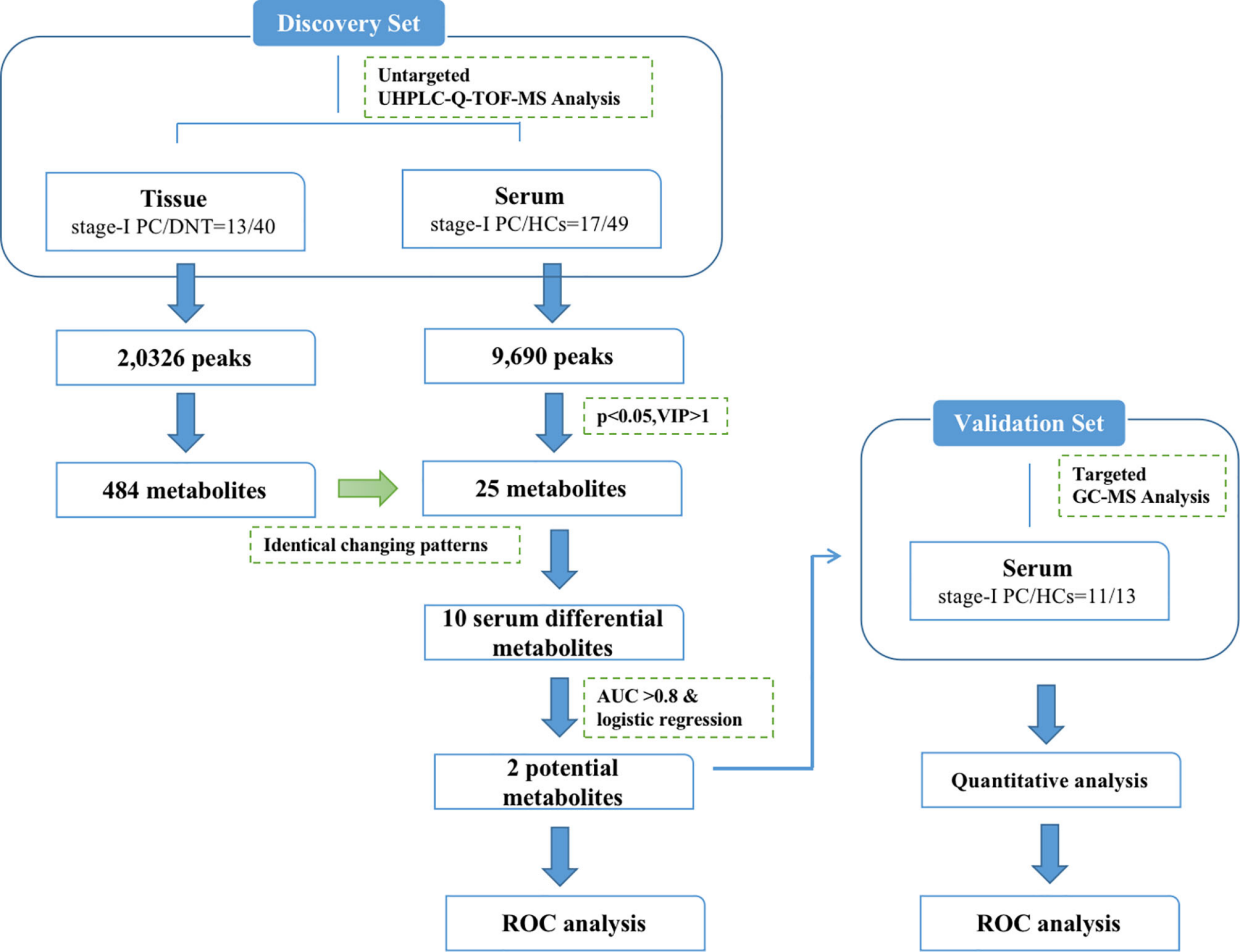
The workflow for the study design and analytical pipeline.

Clinical characteristics for these participants are shown in **Table 1**. There were 90 serum samples (28 stage-I PDAC and 62 HCs) and 53 tissue samples (13 stage-I PDAC and 40 DNTs), which were prospectively collected and enrolled in this study. In the stage-I PDAC serum samples, the sample numbers of stage IA and IB were 12 and 16, respectively. The tumors in the head/uncinate of the pancreas were 20, in the body/tail were 8. 3 samples were well-differentiated adenocarcinoma, 21 samples were moderate-differentiated adenocarcinoma, 4 samples were poor-differentiated adenocarcinoma. In the tissue samples, there were 13 paired stage-I PDAC tissues and DNTs samples, and 27 unpaired DNTs. The clinical characteristics for stage-I PDAC tissues and DNTs subsets were not significantly different (*P* > 0.05). The numbers of stage IA and IB PDAC tissue sample were 5 and 8, respectively, with the median age of 63 years old. The tumors in the head/uncinate of the pancreas were 9, in the body/tail were 4. 11 samples were moderate-differentiated adenocarcinoma, 2 samples were poor-differentiated adenocarcinoma. No significant difference existed between PDAC serum and tissue samples on clinical indicators. The samples in this study were divided into discovery and validation sets. The discovery set includes 66 serum samples (17 stage-I PDAC and 49 HCs) and 43 tissue samples (13 paired stage-I PDAC tissues and DNTs samples and 27 unpaired DNTs). The validation set includes 24 serum samples (11 stage-I PDAC and 13 HCs). Notably, the age of stage-I PDAC (61.24 ± 7.13) was significantly higher than HCs (52.55 ± 6.61) in the serum samples of discovery set. The workflow of this study was shown in **Figure 1**.

**Table 1:** Clinical characteristics of patients with stage-I pancreatic cancer and healthy controls in training and validation sets.

	Discovery Set						Validation Set		
	Serum			Tissue			Serum		
	Stage-1 P DAC	HC^a^	*p*	Stage-1 P DAC	DNT^b^	*p*	Stage-1 P DAC	HC	*p*
N	17	49		13	40		11	13	
Gender									
Male	10	21	0.12	7	23	0.81	6	7	1.00
F emale	7	28		6	17		5	6	
Age	61.24±7.13	52.55±6.6	0.00	63.0±8.95	63.35±8.75	0.76	62.50±9.38	54.61±6.10	0.06
(mean ±S D)		1							
Diabetes									
No	11	49		8		–	8	13	—
T2DM^c^	1	0		0			0	0	
NOD^d^	5	0		5			3	0	
Hypertension									
No	10	35	0.34	10	28	0.74	7	10	0.659
Yes	7	14		3	12		4	3	
Smoking History^e^									
No	6	29	0.091	3	13	0.77	2	4	0.649
Yes	11	20		10	27		9	9	
Tumor stage									
IA	5			5	5		7		
IB	12			8	8		4		
S ite of primary tumor									
Head/Uncinate	12			9			8		
B ody/Tail	5			4			3		
Histologic grade									
W ell	0			0			3		
Moderate	15			11			6		
P oor	2			2			2		
CA19-9(U/ml)									
<37	7	49		3			4	13	
>37	10	0		10			7	0	

^a^HC was healthy controls.

^b^DNT was distal noncancerous tissue.

^c^T2DM was classified as the patients with T2DM two or more years.

^d^NOD was classified as the patients with new-onset diabetes (≦ 2 years duration).

^e^Smoking History was classified as the patients with more than 1 year of smoking history.

### Untargeted Metabolic Profile of Serum in Stage-I PDAC

After data processing, a total of 9,690 metabolic peaks were obtained from serum samples. In positive ion mode, there were 4,341 identifiable peaks. In negative ion patterns, there were 5,349 identifiable peaks. In the PCA plot, all QC samples were tightly clustered together, which indicated satisfactory analysis stability.

**Figure 2:**
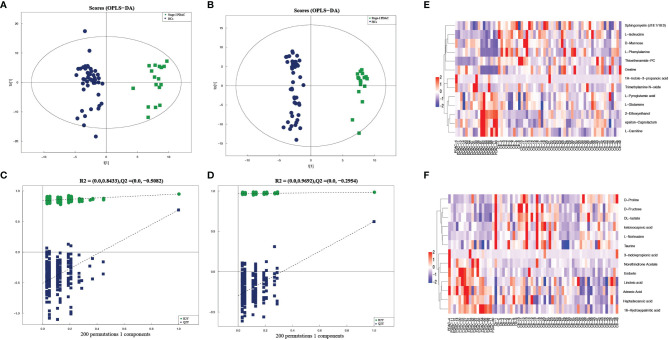
**(A, B)** Serum metabolic profiles resulting from OPLS-DA in positive and negative ion mode. **(C, D)** The statistical validation of the corresponding OPLS-DA models (both in positive and negative ion mode) by permutation tests (200 times). **(E, F)** Heatmaps of prominent differential metabolites from stage-I PDAC versus normal controls in serum samples in positive and negative ion mode.

The OPLS-DA models were performed to analyze the metabolite profile of the two groups. The two groups have significant differences, with satisfactory values of R2Y and Q2 (R2Y = 0.949, Q2 = 0.689 in positive ion mode; R2Y = 0.984, Q2 = 0.621 in negative ion mode) (**Figures 2A, B**). After 200 permutations tests, the reliability of the model and no over-fitting were illustrated (R^2^ = 0.8433, Q^2^ = -0.5082 in positive ion mode; R^2^ = 0.9692, Q^2^ = -0.2954 in negative ion mode) (**Figures 2C, D**). These findings show that the OPLS-DA model could be used to distinguish stage-I PDAC patients from healthy controls. Besides, the univariate test was used to further screen differential metabolites. As a result, given VIP >1.0 and *p* < 0.05, a total of 25 candidate serum metabolites were identified, in which 12 were decreased and 13 were elevated (**Table S1**). The relative quantities of top-differential metabolites were evaluated and presented as heatmaps. Decreased levels of amino acids (isoleucine, phenylalanine, pyroglutamic acid, proline, norleucine, taurine) and elevated levels of fatty acids (linoleic acid, adrenic acid, heptadecanoic acid, 16-hydroxypalmitic acid) were observed in stage-I PDAC (**Figures 2E, F**).

### Untargeted Metabolic Profile of Tissues in Stage-I PDAC

To further verify the metabolic profile of stage-I PDAC, the tissue samples were also assessed using identical analysis. A total of 20,326 metabolic peaks were obtained from tissue samples. In PCA plots, QC samples were clustered, indicating the outstanding stability of the detection condition. In the OPLS-DA models, healthy controls were also separated well from stage-I PDAC patients, with satisfactory values of R2Y and Q2 (**Figures S1A, B**). After 200 permutations tests, the reliability of the model and no over-fitting were also illustrated (**Figures S1C, D**). A total of 484 metabolites were identified, and given VIP > 1.0 and *p* < 0.05, a total of 164 candidate tissue metabolites were further identified. Among them, 126 were decreased and 38 were elevated. The relative quantities of top-differential metabolites were evaluated and presented as heatmaps in both positive and negative ion patterns. Decreased levels of amino acids and elevated levels of fatty acids were also observed (**Figures S1E, F**).

### Establishment of Potential Metabolic Biomarkers for Stage-I PDAC

We identified ten serum metabolites that presented the same expression levels as those in tissues. Among them, 2-ethoxyethanol, adrenic acid, and 3-indolepropionic acid showed upward trends. And the levels of isoleucine, phenylalanine, creatine, lactate, fructose, norleucine, and proline showed downward trends. ROC curves were used to evaluate the diagnostic performance of the biomarkers, and the variables with an AUC > 0.8 were selected (**Table 2**).

**Table 2:** List of potential metabolites presented the same expression levels both in serum and tissue samples of stage-I PDAC patients.

ESI mode	Metabolites	Serum					Tissue			
		AUC	VIP	Fold Change	p-value	Up/down	VIP	Fold Change	p-value	Up/down
+	L-Isoleucine	0.867	1.945	0.679	<0.001	↓	0.801	0.293	0.0240	↓
+	2-Ethoxyethanol	0.719	1.698	1.539	0.001	↑	0.606	1.056	0.781	↑
+	L-Phenylalanine	0.717	1.156	0.604	0.004	↓	4.914	0.130	<0.001	↓
+	Creatine	0.661	1.297	0.632	0.039	↓	0.107	0.527	0.304	↓
–	Adrenic Acid	0.813	1.993	1.533	<0.001	↑	0.402	1.374	0.171	↑
–	DL-lactate	0.794	2.259	0.715	0.001	↓	0.049	0.731	<0.001	↓
–	D-Fructose	0.738	5.546	0.707	0.011	↓	0.647	0.248	0.001	↓
–	L-Norleucine	0.761	3.587	0.697	0.022	↓	4.815	0.150	<0.001	↓
–	3-Indolepropionic acid	0.535	6.715	5.757	0.029	↑	0.260	1.083	0.523	↑
–	D-Proline	0.720	1.323	0.710	0.036	↓	0.378	0.545	0.003	↓

Finally, a total of 2 metabolites showed excellent performance, including adrenic acid and isoleucine. Adrenic acid presented an upward trend, with an AUC score of 0.813, a sensitivity of 76.5%, and a specificity of 75.5%, respectively. Isoleucine presented a downward trend, with an AUC score of 0.867, a sensitivity of 79.6%, and a specificity of 94.1%, respectively.

### Diagnostic Performance of Metabolites Model and Verification of Differential Metabolites by Targeted Analysis in Independent Cohorts

**Figure 3:**
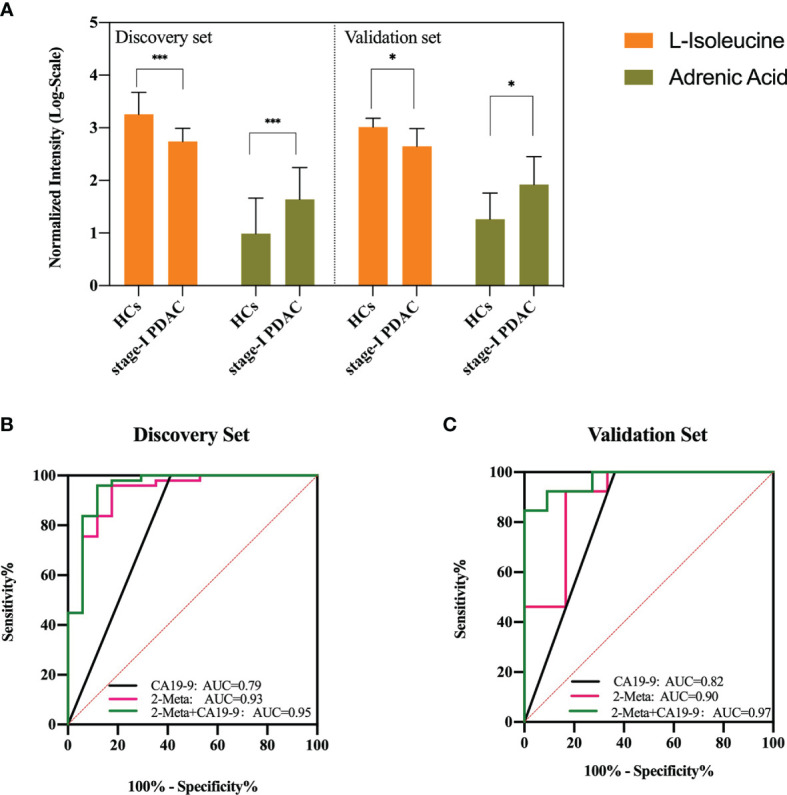
**(A)** Serum concentrations of defined potential biomarkers in the discovery and validation sets, respectively. * represents p<0.05, *** represents p<0.001. **(B, C)** ROC curves of the two-metabolite model, CA19-9, and the combination of two-metabolite model and CA19-9 in the discovery set and validation set. AUC, area under the curve. The red line indicates the metabolite model, black line indicates CA19-9 (cutoff = 37 U/ml), and green line indicates the metabolite model plus CA19-9.

Then the 2-metabolites-based logistic model was constructed, and there were no collinearity and correlation between the two differential metabolites. Compared with the individual metabolite, the AUC value, sensitivity, and specificity of the combinational metabolites were increased to 0.93 (95% CI 0.86 - 1.00), 82.4%, and 95.9%. In the clinic, the standard upper value of serum CA19-9 was 37 U/ml. In our study, the AUC value of CA19-9 was 0.79 (95% CI 0.64 - 0.95), with a cutoff of 37 U/ml. This metabolites model had higher diagnostic performance (AUC 0.93 versus 0.79) and specificity (95.9% versus 58.8%) than those of CA19-9 alone in the distinguishing stage-I PDAC from HCs (**Figure 3B**).

The external validation set, another independent serum sample, including stage-I PDAC (n = 11) and HCs (n = 13). The results showed that adrenic acid and isoleucine were still significantly (p < 0.05) different between the two groups in the validation set, and showed similar variable tendencies with those of the discovery set (**Figure 3A**). The AUC, sensitivity, and specificity of the metabolites model were 0.90 (95% CI 0.72-1.00), 83.3%, and 92.3%, respectively. The AUC value of CA19-9 was 0.82 (95% CI 0.63 - 1.00), with a cutoff of 37 U/ml. This metabolites model had higher diagnostic performance (AUC 0.90 versus 0.82) and specificity (92.3% versus 63.6%) than those of CA19-9 in the distinguishing stage-I PDAC from HCs. We also assessed the performance of a combined model (2 potential metabolites and CA19-9) that discriminates stage-I PDAC from HCs. In our discovery set, the combined model showed a better predictive performance than metabolites model or CA19-9 alone (AUC = 0.95 vs 0.93, 0.79), with a sensitivity of 95.92%, and a specificity of 88.24%, respectively (**Figure 3B**). In our validation set, the combined model also showed a better predictive performance than metabolites model or CA19-9 alone (AUC = 0.97 vs 0.90, 0.82), with a sensitivity of 84.62%, and a specificity of 100.00%, respectively (**Figure 3C**).

### The Effect of Age and Gender on Metabolites

There was an insufficient age-matching and differences in gender between stage-I PDAC and HCs. Therefore, to examine the influence of age and gender on the two potential metabolites, a multivariate logistic model based on the age, gender, and 2 metabolites was constructed. The result showed that adrenic acid, isoleucine, and age were independently associated with stage-I PDAC (*P* < 0.05), and gender was not a significant risk factor (*P* > 0.05) (**Table S2**). Then, the discovery cohort was divided into two groups based on the median age of all subjects (54 years) and gender, respectively. All metabolites showed the same significantly change patterns (*P* < 0.05) in every subcohort as seen in the total population. Moreover, the metabolite model also showed high diagnostic performance in both young and old subcohorts (AUC=0.970, 0.895), and both male and female subcohorts (AUC=0.959, 0.914) (**Table S3**). Additionally, we assessed the correlation between the independent risk factors (age and 2 metabolites) in total population, PDAC subcohort, and HCs subcohort, respectively. As shown in **Figure S2**, there were no significant correlations between age and the expression of two metabolites and age in our three subset (*P* > 0.05). Overall, these results indicate that there were no marked effects of age and gender on the diagnostic performance of the metabolites model.

### Enrichment and Pathways Analysis

Based on the serum and tissue differential metabolites data between stage-I PDAC patients and HCs, a total of 11 pathways were identified as the significant (*p*<0.05) perturbed metabolic pathways in both serum and tissue. Among them, central carbon metabolism in cancer and ABC transporters were the most significantly perturbed metabolic pathways (**Figures S3A, B**).

## Discussion

As analysis technology advances, metabolomics has become a powerful tool in new biomarkers discovery and pathogenesis exploring for PDAC (13, 15). To the best of our knowledge, this is the first study to use HPLC-QTOF-MS untargeted analysis combined with GC-TOF-MS targeted analysis to identify differential putative metabolites for PDAC. In this study, based on the untargeted metabolomics platform, we first identified 25 serum differential metabolites between stage-I PDAC and HCs. To further confirm these serum metabolites to enable the specific diagnosis for PDAC, pancreatic cancer and adjacent noncancerous tissue samples were also used. And a total of 10 serum metabolites expression levels showed the same trends as those in tissues. Based on these potential metabolites, a 2-metabolites-model, including adrenic acid and L-isoleucine was constructed and validated by targeted metabolic analysis. Compared with the conventional biomarker, CA19-9, this metabolic model had the more excellent diagnostic capacity to distinguish stage-I PDAC and HCs.

Most of the previous studies have little concern about the metabolic biomarkers for PDAC in the early stage, especially for stage-I PDAC. Ariadna et al. (16) identified a panel of nine serum biomarkers for distinguishing PDAC and normal controls using untargeted analysis. The panel included phospholipids, fatty acids, bile acids, and amino acids, with an AUC of 0.992. Sandra et al. (17) identified four significant metabolites with lower levels in serum of PDAC patients compared with normal controls. The four biomarkers were connected with lipids metabolism, and the associated AUC was 0.962. The predictive performances of these models were relatively high, which may be related to the lack of external validation. Apart from serum, several previous studies have shown the metabolic difference in other types of samples from PDAC, such as urinary, saliva, and tissues (18). Sumit et al. (19) identified and validated a panel of six urinary metabolites for distinguishing PDAC from normal controls, with the AUC of 0.933 in the discovery set and 0.864 in the validation set. However, all PDAC patients in this study were from the same institution, which was a limitation for the clinical significance.

In our study, as an independent biomarker, isoleucine was lower in stage-I PDAC than those of healthy groups, and showed a favorable diagnostic performance (AUC = 0.867). Isoleucine belongs to the branched-chain amino acids (BCAAs) groups. As energy substances involved in the TCA cycle, BCAAs could provide more nutrition for the proliferation of cancer cells (20). Isoleucine for diagnosing PDAC has been reported in many studies (21–23). Zhang et al. (21) used ^1^H NMR analysis to investigate the plasma samples and proposed a biomarker group to distinguish PDAC patients from chronic pancreatitis and normal controls. The panel included isoleucine, and showed a decreased level in PDAC group, which was consistent with our result. However, Mayers et al. (22) observed that the level of isoleucine was elevated in PDAC, which was inconsistent with ours finding. Such a result may be caused by the cohort differences, as not all PDAC individuals were in early-stage in that study. The decreased levels of amino acids may imply the upregulation of the corresponding biosynthetic pathways in cancer (24). In addition, the relationship between diabetes mellitus (DM) and PDAC is complex (25), so the potential screening performance of isoleucine in high-risk individuals is also worth a mention. Suguru et al. (26) have showed that the significantly increased level of isoleucine was associated with PDAC in DM. Michálková et al. (27) used ^1^H NMR metabolomic analysis to discriminate PDAC patients from healthy controls and also from T2DM by plasma samples. Isoleucine was found increased in T2DM, but without statistical significance. However, our control group just included healthy individuals, so the capability of isoleucine in differentiating stage-IPDAC and chronic pancreatitis or DM is worthy of making further validation.

In line with the previous reports (16, 28), the serum level of phenylalanine was downregulated in PDAC patients. Phenylalanine belongs to the aromatic amino acid family, which could reduce the expression of immunosuppressive factors in cancer (29). Proline is one of the nonessential amino acids, which has been shown that it can be used as the main carbon source by pancreatic cancer to maintain mitochondrial function and the TCA cycle (30, 31). A decrease of proline level in PDAC has been reported in several studies, which may indicate the vigorous metabolism of pancreatic cancer cells (27, 32, 33).

Dysregulation of lipid metabolism is another major feature of cancers. In particular, increased *de novo* synthesis of fatty acids plays a key role in the energy supply, biological membranes synthesis, and signal transduction of tumor (34). Adrenic acid, a polyunsaturated fatty acid, was showed significantly upregulated in stage-I PDAC patients in our study. And another study also observed the upregulated level of adrenic acid in the serum of PDAC patients, although it wasn’t included in the final model (16). In our study, adrenic acid as the biomarker of pancreatic cancer is first reported, and the associated AUC value was 0.813. This result indicated that adrenic acid may help distinguish stage-I PDAC patients from healthy controls. Nevertheless, the application value of adrenic acid in stage-I PDAC requires a large sample to further validate.

To date, there were many “omics” methods used for biomarker identification, such as genomics, transcriptomics, proteomics, and metabolomics. But due to signal amplification in the direction of genome-proteome-metabolome, unique metabolites are closely related to the phenotype and allow more precise and earlier diagnosis for cancer (12). Apart from “omics” techniques, several studies have designed various stochastic sensors, which could be used for the recognition and quantification of cancer biomarkers based on a prior information (35–38). Compared with stochastic sensors, untargeted metabolomics offers an unbiased means to measure the broadest range of metabolites without priori information, which could reveal novel and unique perturbations in cancer (14). HPLC-QTOF-MS is an untargeted metabolomics platform, having the capacity to detect thousands of metabolites, and GC-TOF-MS is a targeted metabolomics platform that has higher separation and quantitation ability (13). In our study, we used an untargeted platform on multi-type samples from multi-institutional stage-I PDAC to identify the potential differential metabolites which presented the same expression levels both in serum and in tissue. Additionally, we used a more precise targeted metabolic platform for external validation. These methods could guarantee our results couldn’t be biased by the small sample size of stage-I PDAC.

Our study has some limitations. First, samples size of stage-I PDAC patients was small. Given the fact that a majority of PDAC patients are diagnosed at an advanced stage, it’s difficult to obtain enough samples of stage-I PDAC. Therefore, future prospective studies with larger stage-I PDAC cohorts are needed to validate our results. Second, there are insufficient age-matching and differences in gender between stage-I PDAC and HCs in our study, but it also illustrates the age and gender tendency of PDAC. Considering that there were no marked effects of age and gender on the selected metabolites, age and gender were not included in our model. Third, our control group was just comprised healthy individuals, so future studies should recruit some individuals with at-risk populations (eg, chronic pancreatitis, the new-onset diabetes, and maybe also as advanced diabetes) into control group, aiming to explore the clinical diagnostic potential of this metabolite model. Fourth, we screened a total of 10 differential serum metabolites, which presented the same expression level as those in tissues. However, for those, whose expression levels were inconsistent with those in tissue, the metabolism mechanisms and diagnostic values deserve future research. Lastly, future studies will include the prognostic information of PDAC patients to evaluate the clinical value of the selected metabolic model in this study, and will combine with the genomic and proteomic analysis to further explore the mechanisms of the metabolite changes.

In summary, our study identified a novel model of two serum metabolites that could be used for the early detection of stage-I PDAC. Although this study is still in its infancy, it could lay the foundation for the future development of metabolomics for stage-I PDAC detection. And the ultimate aim of these studies is to improve the accuracy of diagnosis for PDAC in the early stage, which has great significance for the prognosis.

## Data Availability Statement

The original contributions presented in the study are included in the article/**Supplementary Material**. Further inquiries can be directed to the corresponding authors.

## Ethics Statement

The studies involving human participants were reviewed and approved by the First Affiliated Hospital of Nanjing University of Chinese Medicine. The patients/participants provided their written informed consent to participate in this study.

## Author Contributions

YC and RZ contributed equally to this work. YC and RZ designed the study, performed the data analysis, and drafted the manuscript. KG and SR performed the project administration. YZ collected the data. XC modified the manuscript. ZQW provided funding and designed this study. All authors contributed to the article and approved the submitted version.

## Funding

This work was supported by the National Natural Sciences Foundation of China Grant (grant number 81771899, 82171925) and Postgraduate Research & Practice Innovation Program of Jiangsu Province (grant number KYCX21_1702).

## Conflict of Interest

The authors declare that the research was conducted in the absence of any commercial or financial relationships that could be construed as a potential conflict of interest.

## Publisher’s Note

All claims expressed in this article are solely those of the authors and do not necessarily represent those of their affiliated organizations, or those of the publisher, the editors and the reviewers. Any product that may be evaluated in this article, or claim that may be made by its manufacturer, is not guaranteed or endorsed by the publisher.
