# Supplementary material for: Potential Metabolite Biomarkers for Early Detection of Stage-I Pancreatic Ductal Adenocarcinoma
Source: Front Oncol. 2022 Jan 19;11:744667. doi: 10.3389/fonc.2021.744667 (PMC8807510; doi:10.3389/fonc.2021.744667)
Supplement: Supplementary file 1 [file DataSheet_1.docx]

| ESI  mode | Serum Metabolites |  |  |  |
| --- | --- | --- | --- | --- |
|  |  | VIP | Fold Change | *P*-value |
| + | L-Isoleucine | 1.945 | 0.679 | <0.001 |
| + | 2-Ethoxyethanol | 1.698 | 1.539 | 0.001 |
| + | L-Phenylalanine | 1.156 | 0.604 | 0.004 |
| + | L-Carnitine | 2.461 | 1.363 | 0.001 |
| + | Thioetheramide-PC | 2.019 | 0.495 | 0.009 |
| + | Sphingomyelin (d18:1/18:0) | 1.577 | 0.797 | 0.005 |
| + | L-Glutamine | 1.329 | 1.198 | 0.013 |
| + | L-Pyroglutamic acid | 1.156 | 0.604 | 0.004 |
| + | D-Mannose | 1.128 | 0.846 | 0.049 |
| + | 1H-Indole-3-propanoic acid | 1.061 | 4.097 | 0.047 |
| + | epsilon-Caprolactam | 1.050 | 1.361 | <0.001 |
| + | Creatine | 1.297 | 0.632 | 0.039 |
| - | Linoleic acid | 14.435 | 1.369 | 0.003 |
| - | ketoisocaproic acid | 3.975 | 0.611 | 0.018 |
| - | Embelin | 1.764 | 2.313 | <0.001 |
| - | Taurine | 1.715 | 0.829 | 0.027 |
| - | Norethindrone Acetate | 1.442 | 1.879 | <0.001 |
| - | Heptadecanoic acid | 1.077 | 1.258 | 0.006 |
| - | 3-Indolepropionic acid | 6.715 | 5.757 | 0.029 |
| - | Adrenic Acid | 1.993 | 1.533 | <0.001 |
| - | DL-lactate | 2.259 | 0.715 | 0.001 |
| - | D-Fructose | 5.546 | 0.707 | 0.011 |
| - | L-Norleucine | 3.587 | 0.697 | 0.022 |
| - | 3-Indolepropionic acid | 6.715 | 5.757 | 0.029 |
| - | D-Proline | 1.323 | 0.710 | 0.036 |

**Table S1.** List of serum metabolites with significantly different levels between stage-I PDAC patients and HCs.

**Table S2.** Multivariable Logistic regression analysis in total population.

|  | *P* | OR |
| --- | --- | --- |
| Age | 0.012 | 1.425 |
| Gender | 0.378 | 2.834 |
| Adrenic acid | 0.015 | 14.125 |
| Isoleucine | 0.014 | 0.146 |

**Table S3.** Univariate Analysis of Metabolites within Age and Gender Specific Cohorts.

|  | Age | | | | | | |  | Gender | | | | | | |
| --- | --- | --- | --- | --- | --- | --- | --- | --- | --- | --- | --- | --- | --- | --- | --- |
|  | Young (≦54 years age) | | |  | Old ( >54 years age) | | |  | Female | | |  | Male | | |
|  | FC | *P*-value | AUC |  | FC | *P*-value | AUC |  | FC | *P*-value | AUC |  | FC | *P*-value | AUC |
| L-Isoleucine | 0.674 | 0.001 | 0.869 |  | 0.681 | 0.001 | 0.870 |  | 0.696 | 0.004 | 0.842 |  | 0.656 | <0.001 | 0.914 |
| Adrenic Acid | 1.613 | 0.002 | 0.867 |  | 1.438 | 0.002 | 0.770 |  | 1.715 | 0.002 | 0.865 |  | 1.367 | 0.009 | 0.788 |
| 2-Metabolites model | - | - | 0.970 |  | - | - | 0.895 |  | - | - | 0.959 |  | - | - | 0.914 |

**
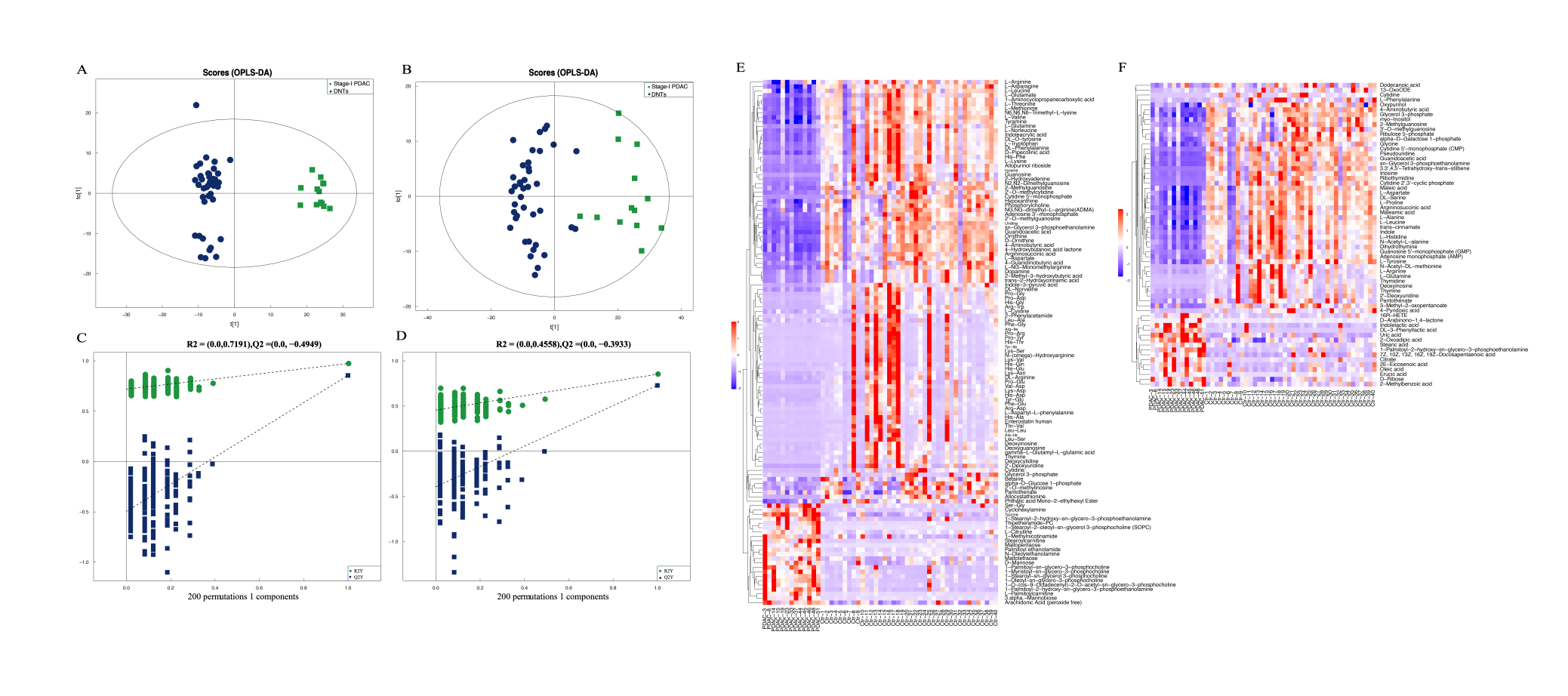
**

**Figure S1.** (**A, B**) Tissue metabolic profiles resulting from OPLS-DA in positive and negative ion mode. (**C, D**) The statistical validation of the corresponding OPLS-DA models（both in positive and negative ion mode）by permutation tests (200 times). (**E，F**) Heatmap of the prominent differential metabolites from stage-I PDAC tissues versus DNT in positive and negative ion mode.

**
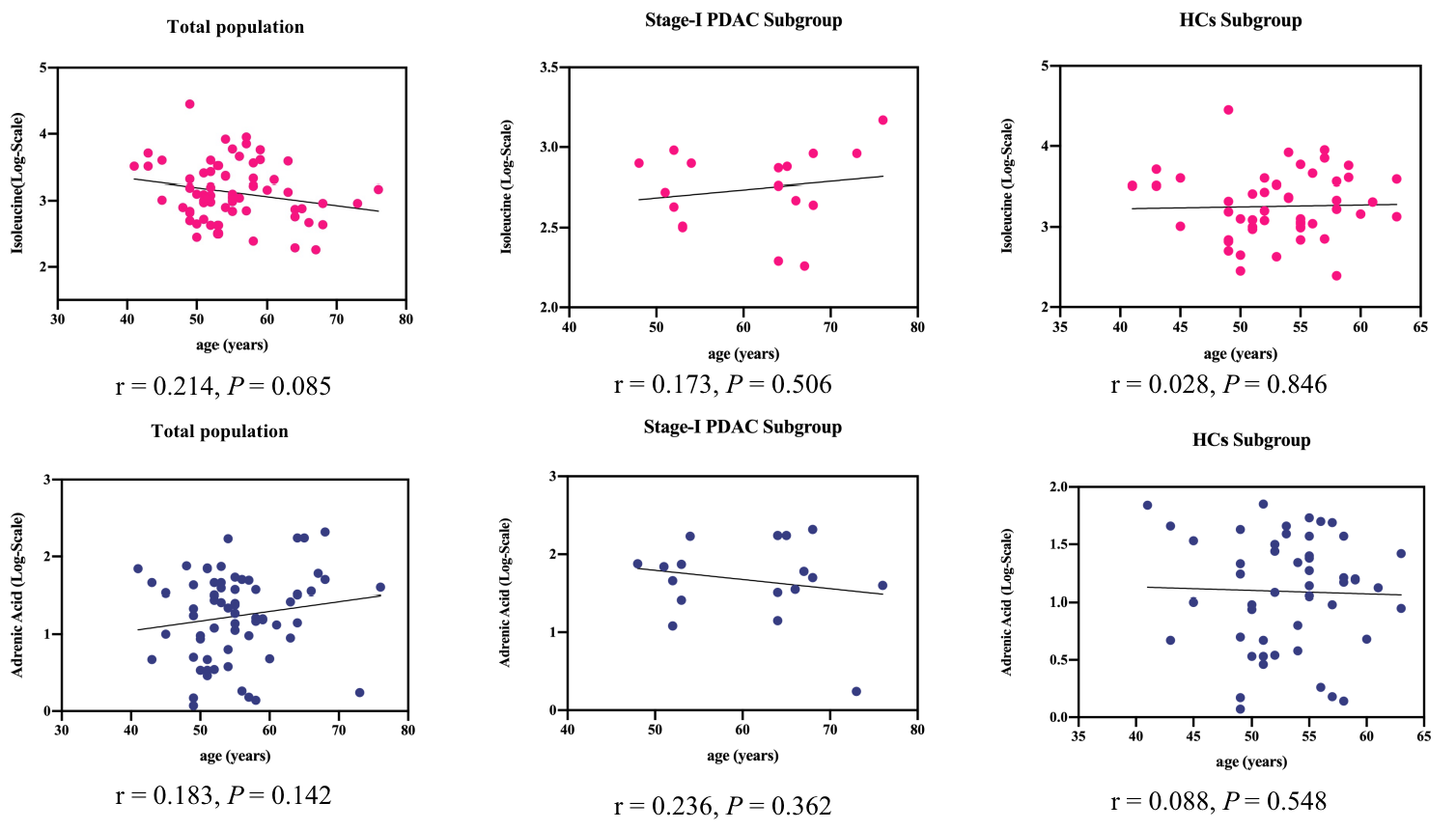
**

**Figure S2.** The scatter plot of age and metabolites in three groups. Red plots indicates L-isoleucine, and blue plots indicates adrenic acid.

**
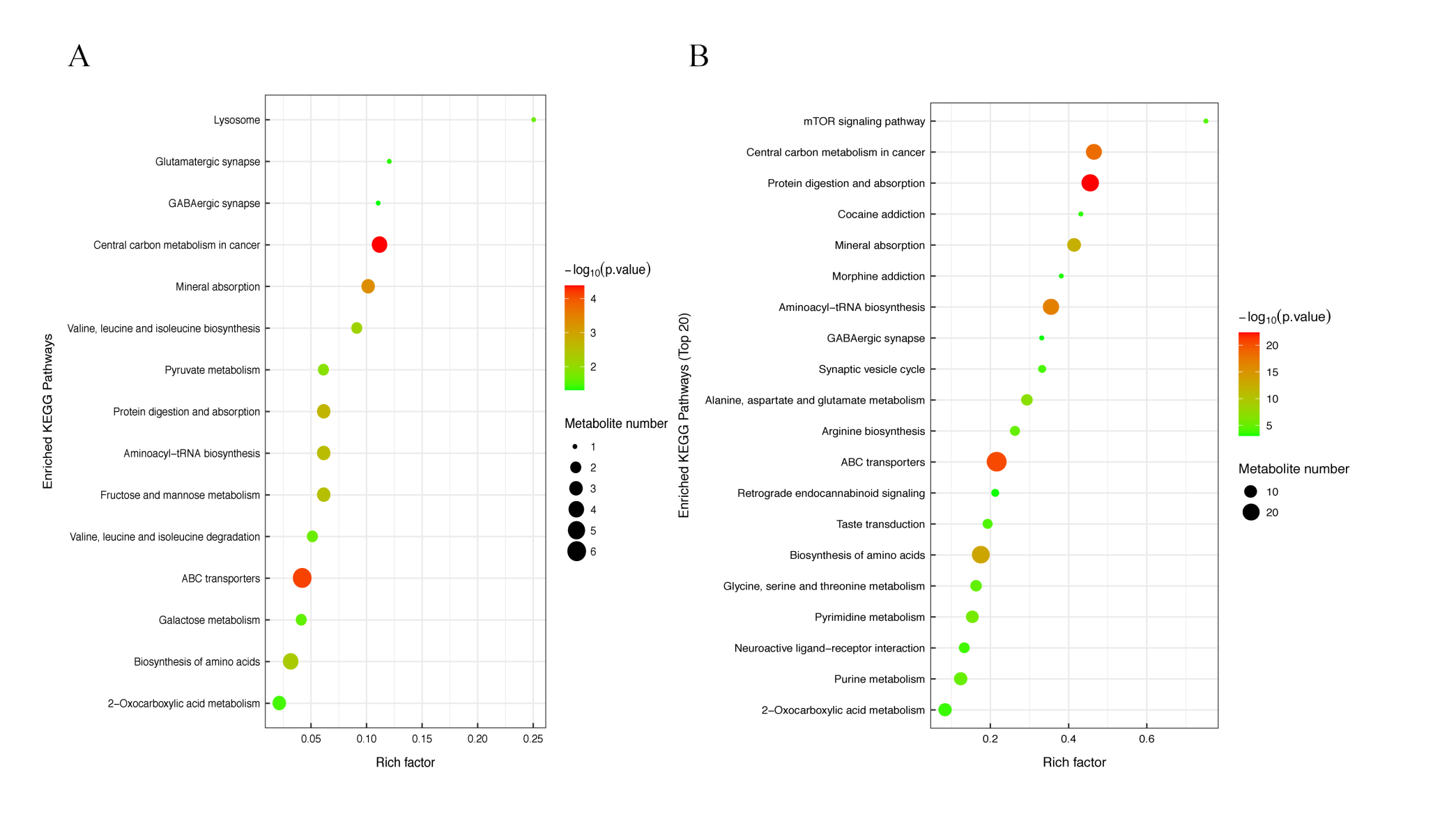
**

**Figure S3.** Results of pathway analysis of serum (**A**) and tissue (**B**) metabolomics data.
